# Xeno-Free Biomimetic ECM Model for Investigation of Matrix Composition and Stiffness on Astrocyte Cell Response

**DOI:** 10.3390/jfb14050256

**Published:** 2023-05-05

**Authors:** Bayan M. Saleh, Ayda Pourmostafa, Nashaita Y. Patrawalla, Vipuil Kishore

**Affiliations:** Department of Biomedical and Chemical Engineering and Sciences, Florida Institute of Technology, Melbourne, FL 32901, USA

**Keywords:** extracellular matrix, hyaluronic acid, collagen, hydrogel, astrocytes, Alzheimer’s disease

## Abstract

Astrocytes, highly specialized glial cells, play a critical role in neuronal function. Variations in brain extracellular matrix (ECM) during development and disease can significantly alter astrocyte cell function. Age-related changes in ECM properties have been linked to neurodegenerative diseases such as Alzheimer’s disease. The goal of this study was to develop hydrogel-based biomimetic ECM models with varying stiffness and evaluate the effects of ECM composition and stiffness on astrocyte cell response. Xeno-free ECM models were synthesized by combining varying ratios of human collagen and thiolated hyaluronic acid (HA) crosslinked with polyethylene glycol diacrylate. Results showed that modulating ECM composition yielded hydrogels with varying stiffnesses that match the stiffness of the native brain ECM. Collagen-rich hydrogels swell more and exhibit greater stability. Higher metabolic activity and greater cell spreading was observed in hydrogels with lower HA. Soft hydrogels trigger astrocyte activation indicated by greater cell spreading, high GFAP expression and low ALDH1L1 expression. This work presents a baseline ECM model to investigate the synergistic effects of ECM composition and stiffness on astrocytes, which could be further developed to identify key ECM biomarkers and formulate new therapies to alleviate the impact of ECM changes on the onset and progression of neurodegenerative diseases.

## 1. Introduction

Alzheimer’s disease (AD) is a highly pervasive neurodegenerative disease that causes dementia and cognitive decline [1]. Despite significant research, a reliable method for the treatment of AD is lacking due to poor understanding of the underlying mechanisms that trigger the onset and progression of the disease [2,3]. Therefore, there is an urgent need for new approaches to understand the mechanisms of AD progression towards the development of novel biomarkers and therapeutic strategies for early diagnosis and treatment of this deadly disease [4].

The extracellular matrix (ECM) microenvironment is a tissue-specific three-dimensional network that not only acts as a physical support to the cells but is also known to provide the essential cues to control and direct cell fate [5,6]. Brain ECM is predominantly made up of hyaluronic acid (HA) and proteoglycans, and low levels of link and fibrous proteins which together control brain cell survival, axonal guidance, and maintenance of synaptic plasticity [7,8]. Variations in brain ECM properties during development and disease have been shown to alter cell function, both positively by promoting tissue remodeling, and negatively by disrupting tissue homeostasis [9]. Recent work on AD progression suggests a causal relationship between age-related changes in the properties of the brain ECM and the onset of AD [10,11]. Development of in vitro brain ECM models that mimic the physicochemical properties of the native tissue can enable investigation of the impact of changes in ECM properties on cell function towards a better understanding of the role of the ECM in neurodegenerative diseases such as AD.

Astrocytes are highly specialized multifunctional glial cells that play a critical role in overall brain homeostasis [12]. In a process termed ‘reactive astrogliosis’, astrocytes are known to change phenotype from a quiescent state to reactive state in response to pathophysiological changes in the brain because of trauma or disease [13]. Prior work by Placone et al. reported on a novel 3D matrix composed of collagen, HA and Matrigel and showed that astrocytes maintain their native quiescent phenotype as evidenced by low levels of glial fibrillary acid protein (GFAP) expression [14]. In a separate study, Jimenez-Vergara et al. employed multicomponent interpenetrating polymer networks (mIPNs) and reported that a decrease in HA content resulted in an increase in reactive phenotype markers in astrocytes (i.e., GFAP) [15]. Hu et al. developed hybrid hydrogels composed of collagen type I and alginate with dynamic crosslinking feasibility and showed that change in matrix stiffness can impact astrocyte activation and phenotype [16]. While the impact of ECM properties on astrocytes has been previously reported, studies that investigate the synergistic effects of changes in ECM composition and ECM stiffness on astrocyte behavior are lacking. Additionally, prior studies employed low HA concentrations and animal-derived collagen which may not be clinically relevant. The goal of the current study was to develop xeno-free biomimetic ECM models with tunable stiffness and systematically investigate the effects of changing ECM composition, ECM stiffness, and their interplay on astrocyte cell response.

Hydrogel-based ECM models with varying stiffnesses were prepared by combining different ratios of human skin-derived collagen type I and thiolated HA together with a fixed amount of polyethylene glycol diacrylate (PEGDA) crosslinker. Physical characterization studies entailed the assessment of compressive moduli, stability, and surface microstructure of the hydrogel-based ECM models. Normal human astrocytes (NHA) were encapsulated within the hydrogels and the synergistic effects of ECM composition and stiffness on astrocyte cell morphology, proliferation, and phenotype was investigated. Outcomes of this work can pave the way for further development and the use of biomimetic hydrogel-based ECM models for the identification of key ECM factors that drive AD, and open previously unexplored prospects for therapeutic intervention and cures.

## 2. Materials and Methods

### 2.1. Materials

Human skin-derived collagen type I (HumaDerm) was purchased from Humabiologics (Phoenix, AZ, USA). Thiolated HA (HyStem) and poly(ethylene glycol) diacrylate (PEGDA) were purchased from Advanced Biomatrix (San Diego, CA, USA). NHAs were purchased from Lonza (Morristown, NJ, USA). Primary antibodies (GFAP, ALDH1L1) were purchased from Abcam (Boston, MA, USA). The FITC-labeled secondary antibody was purchased from Jackson ImmunoResearch (Westgrove, PA, USA). All other chemicals and reagents were purchased from Fisher Scientific (Waltham, MA, USA) unless stated otherwise.

### 2.2. Preparation of Xeno-Free Biomimetic Hydrogel-Based ECM Models

Two different concentrations of collagen type I (6 mg/mL or 12 mg/mL) and HA (6 mg/mL or 12 mg/mL) were used with a constant PEGDA concentration (5 mg/mL) to make hydrogel-based ECM models of four different compositions of 6-6-5, 6-12-5, 12-6-5, and 12-12-5 (Col-HA-PEGDA; Table 1). At first, the collagen solution of the desired concentration was neutralized using 8:1:1 ratio of collagen type I, 0.1 N NaOH, and 10x PBS. To this, desired concentrations of HA and PEGDA were added at a volume ratio of 4:4:1 (collagen:HA:PEGDA), and the solution was extruded into rubber washers (7.5 mm diameter, 2.5 mm height) and incubated at 37 °C for 90 min to induce gelation.

### 2.3. Mechanical Assessment of ECM Hydrogels

A Discovery series HR-30 rheometer (TA Instruments, New Castle, DE, USA) calibrated in DMA mode was used for uniaxial compression testing (N = 10 hydrogels/group) to determine the compressive modulus of ECM hydrogels. Briefly, hydrogel-based ECM models were submerged in ultrapure water for 30 s and placed on a 20 mm stainless steel sandblasted stage on the rheometer. Following this, the hydrogels were gently blotted using a KimWipe to remove the excess water and compressed up to 60% hydrogel thickness at a steady loading rate of 10 µm/s. Stress–strain curves for each hydrogel were generated to compute the compressive modulus using the 0–10% strain region.

### 2.4. Assessment of Swelling Capacity and Stability of ECM Hydrogels

Swelling studies were conducted to determine the water absorption capacity of hydrogel-based ECM models (N = 8 hydrogels/group). At first, the initial weight of the hydrogels was measured (W_0_). Following this, the hydrogels were incubated in PBS for 14 days at room temperature. Hydrogels were weighed every day to determine the change in weight over time. PBS solution was replenished daily. Swelling ratio was calculated by taking the ratio of the change in weight of the hydrogel between day 0 (W_0_) and day 1 (W_1_) to the initial weight at day 0 (Equation (1)).

Swelling Ratio (%) = (W_1_ − W_0_)/W_0_ × 100 (1)

For stability assessment, the hydrogels were incubated in PBS for 1 day to allow initial water uptake. Residual mass was calculated based upon the change in weight of the hydrogels from day 1 (W_1_) to the weight at day 7 and day 14 (W_7,14_) (Equation (2)).

Residual Mass (%) = W_7,14_/W_1_ × 100 (2)

### 2.5. Assessment of Surface Microstructure of ECM Hydrogels

Scanning electron microscopy (SEM) was performed to examine the effects of changes in ECM composition on the surface microstructure of hydrogel-based ECM models (N = 3 hydrogels/group). Post fabrication, hydrogels were frozen at −80 °C for 4 h followed by lyophilization for 24 h. Lyophilized samples were mounted onto a stub, sputter coated with gold, and examined under SEM (JEOL JSM-6380LV, Peabody, MA, USA).

### 2.6. Cell Culture and Encapsulation in ECM Hydrogels

NHAs were purchased at passage 1 (P1) from Lonza. Cells were cultured in a 75 cm^2^ flask in growth medium composed of Dulbecco’s Modified Eagle Medium/Nutrient Mixture F-12 (DMEM/F-12) supplemented with 10% FBS and 1% penicillin/streptomycin in 5% CO_2_ at 37 °C for two passages and cryopreserved at passage 3 (P3). Prior to the experiments, cells were removed from cryopreservation and cultured for one additional passage. Passage 4 (P4) cells were used for all experiments. Cells were encapsulated into neutralized collagen type I solution, mixed well, and added to HA and PEGDA (5 × 10^5^ cells/mL). One hundred µL of cell suspension was added into individual wells of an ultralow attachment 96-well plate (Corning), incubated for 60–90 min for gelation, and cultured in growth medium for 14 days.

### 2.7. Cell Metabolic Activity in ECM Hydrogels

Alamar blue assay was used to determine the effects of ECM composition and stiffness on cell metabolic activity of NHA encapsulated in ECM hydrogels (N = 9–12 hydrogels/group/timepoint). At periodic intervals, cells encapsulated in hydrogels were incubated with 10% Alamar blue solution at 37 °C with 5% CO_2_ for 4 h. Following this, 100 µL of the solution was transferred from each well into a new clear bottom 96-well plate (Greiner). Fluorescence was measured using a SpectraMax M2e plate reader (Molecular Devices, San Jose, CA, USA) at an excitation wavelength of 555 nm and emission wavelength of 595 nm. The change in relative fluorescence units (RFU) over time was indicative of cell metabolic activity in the hydrogels.

### 2.8. Qualitative Assessment of Cell Morphology in ECM Hydrogels

To investigate the effects of ECM properties on changes in cell morphology, the cell cytoskeleton was stained using AlexaFluor-488 Phalloidin and imaged using confocal microscopy. Briefly, at periodic intervals, cells encapsulated in hydrogels were fixed in 3.7% formaldehyde for 30 min and permeabilized with 0.05% Triton X-100 in 1x PBS for 15 min. Following this, the hydrogels were incubated in blocking buffer composed of 0.5% bovine serum albumin (BSA) in permeabilization buffer for 30 min. Hydrogels were then washed with 1x PBS and the cell cytoskeleton was stained with AlexaFluor-488 Phalloidin overnight at 4° C. Following this, cell nuclei were stained with 4’,6-diamino-2-phenylindole, dihydrochloride (DAPI) for 5 min. Cells were imaged using a confocal microscope (Eclipse Ti2 series inverted microscope, Nikon, Tokyo, Japan) with z-stack of 1 µm step size for each image and a maximum intensity projection in z direction was applied.

### 2.9. Quantitative Assessment of Cell Morphology in ECM Hydrogels

Quantitative analysis of cell cytoskeleton-stained images was performed to assess the effects of ECM composition and stiffness on NHA morphology. Maximum intensity images were processed using ImageJ (National Institute of health, Bethesda, MD). The number of endpoints, process length, and cell spreading were measured at day 7 and day 14 (N = 6 hydrogels/group/timepoint, N = 3 images/hydrogel/group, with at least N = 3 cells/image). Endpoints were defined by counting the number of extensions on a dendrite. Process length was analyzed by measuring the longest branch length by drawing a line from the center of the nucleus to the furthest endpoint. Cell spreading was measured by tracing the exoskeleton of randomly selected cells and measuring the area.

### 2.10. Evaluation of Cell Phenotype using Immunofluorescence

Immunofluorescence staining was conducted to assess the effect of ECM composition and stiffness on NHA phenotype (N = 6 hydrogels/group/timepoint). ALDH1L1 was used as a quiescent marker and GFAP was used as a reactive marker. At day 7, cell-laden hydrogels were fixed with 3.7% formaldehyde, permeabilized in 0.25% Triton X-100 in 1x PBS and blocked with 1% bovine serum albumin (BSA) buffer. Following this, the hydrogels were incubated with a primary antibody (rabbit-anti human GFAP or rabbit-anti human ALDH1L1) for 24 h in 1% BSA solution at 4 °C. A dilution ratio of 1:100 for GFAP and 1:50 for ALDH1L1 was used. Next, FITC labeled anti-rabbit secondary antibody (1:20–1:50) was added and incubated with the hydrogels for 12 h. Finally, cell nuclei were stained with DAPI and the hydrogels were imaged using a confocal microscope.

### 2.11. Statistical Analysis

Results were expressed as mean ± standard deviation. Each experiment was repeated at least twice to confirm reproducibility. Data were combined from multiple runs to obtain the sample size. Statistical analysis was performed using a one-way ANOVA with a Tukey post-hoc adjustment for pairwise comparisons (JMP PRO 14 Statistical Discovery, SAS, Cary, NC, USA). Statistical significance was set at *p* < 0.05.

## 3. Results

### 3.1. Compressive Modulus of ECM Hydrogels

**Figure 1:**
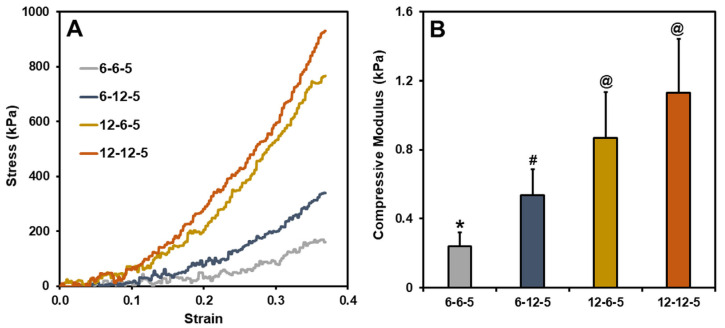
(**A**) Representative stress vs. strain curves for different ECM hydrogels, (**B**) Compressive modulus calculated using the slope of the 0–10% strain region (bars labeled with different symbols are statistically significant (*p* < 0.05)).

Typical stress–strain curves for ECM hydrogels with different compositions of collagen and HA are shown in Figure 1A. The compressive modulus of ECM hydrogels with equal amounts of collagen and HA (6-6-5), determined by taking the slope of the 0–10% strain region, was around 0.2 kPa (Figure 1B). Increasing the amount of HA (6-12-5) resulted in a significant increase (*p* < 0.05) in the compressive modulus of ECM hydrogels to around 0.5 kPa. In a similar vein, increasing the collagen composition (12-6-5) showed a four-fold increase in the compressive modulus at around 0.8 kPa. When comparing the 6-12-5 and 12-6-5 hydrogels, the compressive modulus of collagen-enriched hydrogels (12-6-5) was significantly higher (*p* < 0.05) than the ones with higher HA (6-12-5). ECM hydrogels with the same composition of collagen and HA but with higher concentrations of each of the components (12-12-5) showed a significantly higher (*p* < 0.05) compressive modulus compared to 6-6-5 and 6-12-5 hydrogels. Together, these results indicate that ECM hydrogels with varying stiffness can be synthesized by modulating the collagen and HA composition.

### 3.2. Swelling Ratio and Stability of ECM Hydrogels

**Figure 2:**
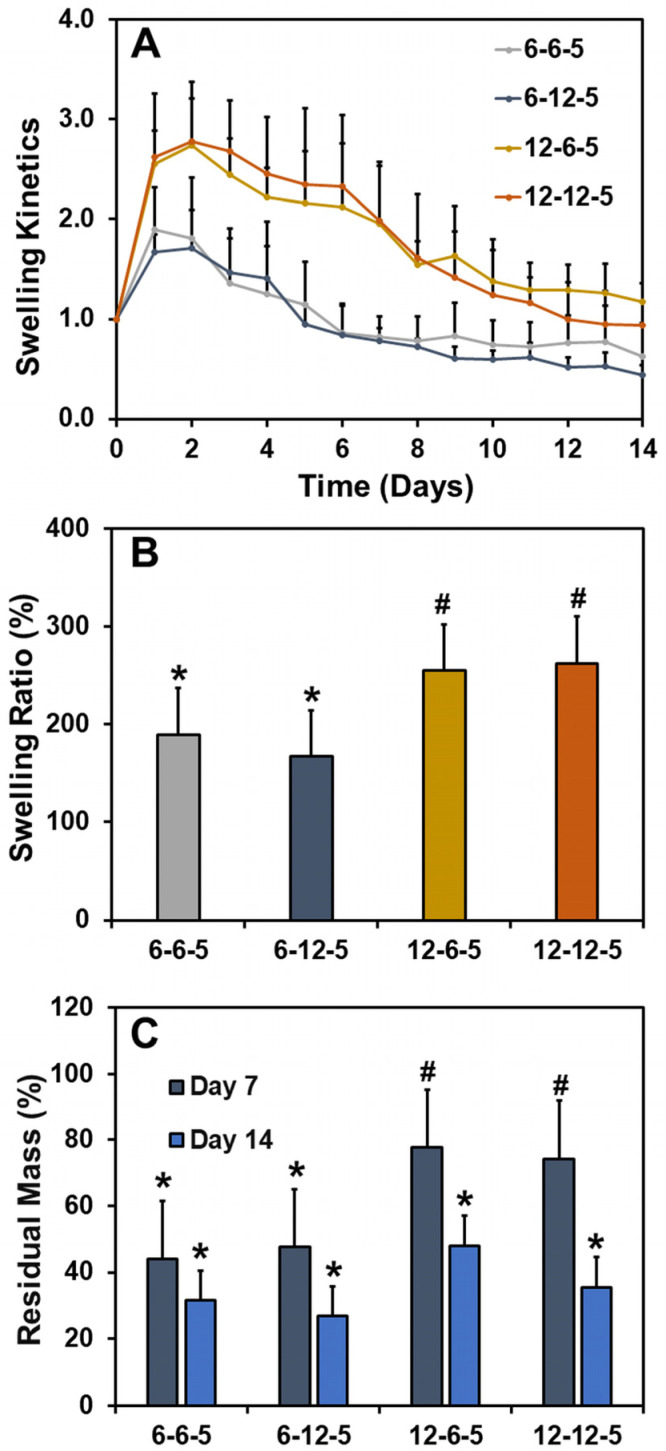
Assessment of swelling ratio and stability of ECM hydrogels. (**A**) Swelling kinetics determined by incubating the hydrogels in 1x PBS and measuring the change in wet weight over time, (**B**) swelling ratio calculated as the ratio of the change in weight of the hydrogel at day 1 to the initial weight at day 0, and (**C**) residual mass calculated as the percentage mass of hydrogel remaining at day 7 and day 14. (Bars labeled with different symbols are statistically significant (*p* < 0.05)).

The effect of ECM properties on the water absorption capacity and stability of hydrogels was determined by performing swelling studies. The swelling kinetics curve revealed that all ECM hydrogels swell initially at day 1 due to water absorption followed by a steady decline in mass over the course of two weeks (Figure 2A). The swelling ratios of ECM hydrogels with 6-6-5 and 6-12-5 compositions were comparable and ranged between 150 and 200% (Figure 2B). Increasing the collagen composition (12-6-5 and 12-12-5) resulted in a significant increase (*p* < 0.05) in water absorption capability of the ECM hydrogels, indicating that hydrogels with a higher collagen content swell more. On the other hand, a change in HA composition had no impact on the swelling ratio of ECM hydrogels. Results for residual mass showed that ECM hydrogels with a higher collagen content were more stable as indicated by ~80% mass of the hydrogel remaining for 12-6-5 and 12-12-5 hydrogels compared to ~40% for 6-6-5 and 6-12-5 hydrogels at day 7 (Figure 2C). Together, these results indicate that increase in collagen composition yield more stable ECM hydrogels.

### 3.3. SEM Assessment of Surface Morphology of ECM Hydrogels

**Figure 3:**
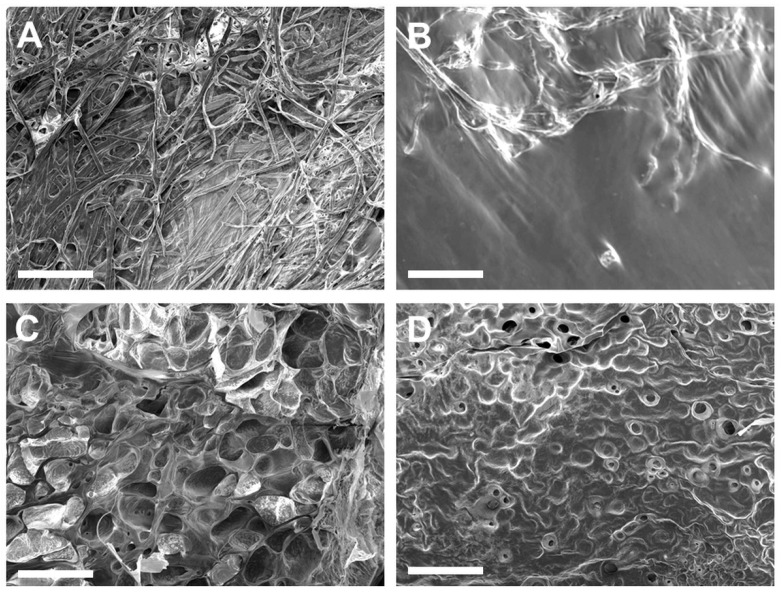
Assessment of surface morphology of ECM hydrogels by SEM: (**A**) 6-6-5, (**B**) 6-12-5, (**C**) 12-6-5, and (**D**) 12-12-5. Scale bar: 500 µm.

ECM hydrogels with the 6-6-5 composition exhibited a fibrous morphology on the surface (Figure 3A). Increasing the collagen content (12-6-5) yielded hydrogels with a porous architecture, with uniform sized pores on the surface of the hydrogel (Figure 3C). Hydrogels with a higher composition of HA (6-12-5, 12-12-5) showed a non-fibrous sheet-like morphology with little to no porosity (Figure 3B,D). The surface texture of the HA-rich hydrogels was different depending on the amount of collagen, wherein 6-12-5 hydrogels showed a smooth surface while 12-12-5 hydrogels exhibited a rougher topography. Together, these results suggest that compositional changes noticeably alter the surface microstructure of ECM hydrogels.

### 3.4. Cell Metabolic Activity in ECM Hydrogels

**Figure 4:**
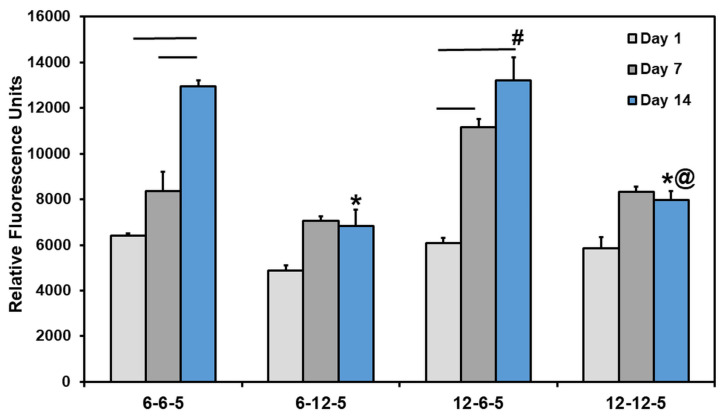
Quantification of change in cell metabolic activity of NHA in ECM hydrogels over time using Alamar blue assay. (* indicates *p* < 0.05 when comparing with 6-6-5 at the same time point, # indicates *p* < 0.05 when comparing with 6-12-5 at the same time point, @ indicates *p* < 0.05 when comparing with 12-6-5 at the same time point. Horizontal lines connecting bars indicate *p* < 0.05 over time within the same group).

Results from the Alamar blue assay showed that the cell metabolic activity of NHA encapsulated in ECM hydrogels increased significantly over time in 6-6-5 and 12-6-5 hydrogels (Figure 4). However, ECM hydrogels with a higher HA composition (6-12-5 and 12-12-5) showed a marginal increase in cell metabolic activity from day 1 to day 14 which was not statistically significant. More importantly, an increase in HA composition resulted in a significant decrease (*p* < 0.05) in cell metabolic activity as indicated by lower RFU measurements for 6-12-5 and 12-12-5 hydrogels compared to 6-6-5 and 12-6-5 hydrogels. When comparing ECM hydrogels with the same composition of collagen and HA (i.e., 6-6-5 and 12-12-5), cell metabolic activity was significantly higher in softer hydrogels (6-6-5) compared to stiffer hydrogels (12-12-5). Together, these results indicate that ECM hydrogels with a lower HA composition and a softer matrix stiffness better support NHA viability and growth.

### 3.5. Qualitative and Quantitative Analyses of Cell Cytoskeleton Staining

**Figure 5:**
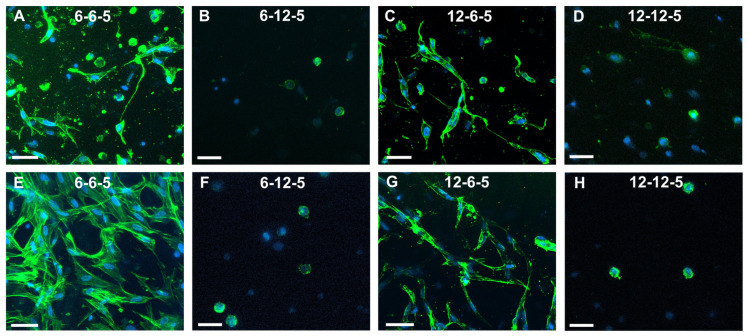
Assessment of NHA morphology via cytoskeleton staining using Alexa Fluor-488 Phalloidin at day 7 (**A**–**D**) and day 14 (**E**–**H**). (**A**,**E**) 6-6-5, (**B**,**F**) 6-12-5, (**C**,**G**) 12-6-5, and (**D**,**H**) 12-12-5 hydrogels. Scale bar: 50 µm.

Confocal microscopy of astrocytes cultured in 6-6-5 hydrogels showed a combination of cells with a round and spread morphology at day 7 (Figure 5A). Cell spreading visibly increased with time, and by day 14 most of the cells encapsulated in 6-6-5 hydrogels were well spread (Figure 5E). Astrocytes encapsulated in collagen-rich hydrogels (12-6-5) showed a spread morphology at both day 7 and day 14 (Figure 5C,G). Increasing the HA content resulted in the cessation of cell spreading as evidenced by the round morphology of astrocytes in 6-12-5 and 12-12-5 hydrogels at both time points (Figure 5B,D,F,H).

**Figure 6:**
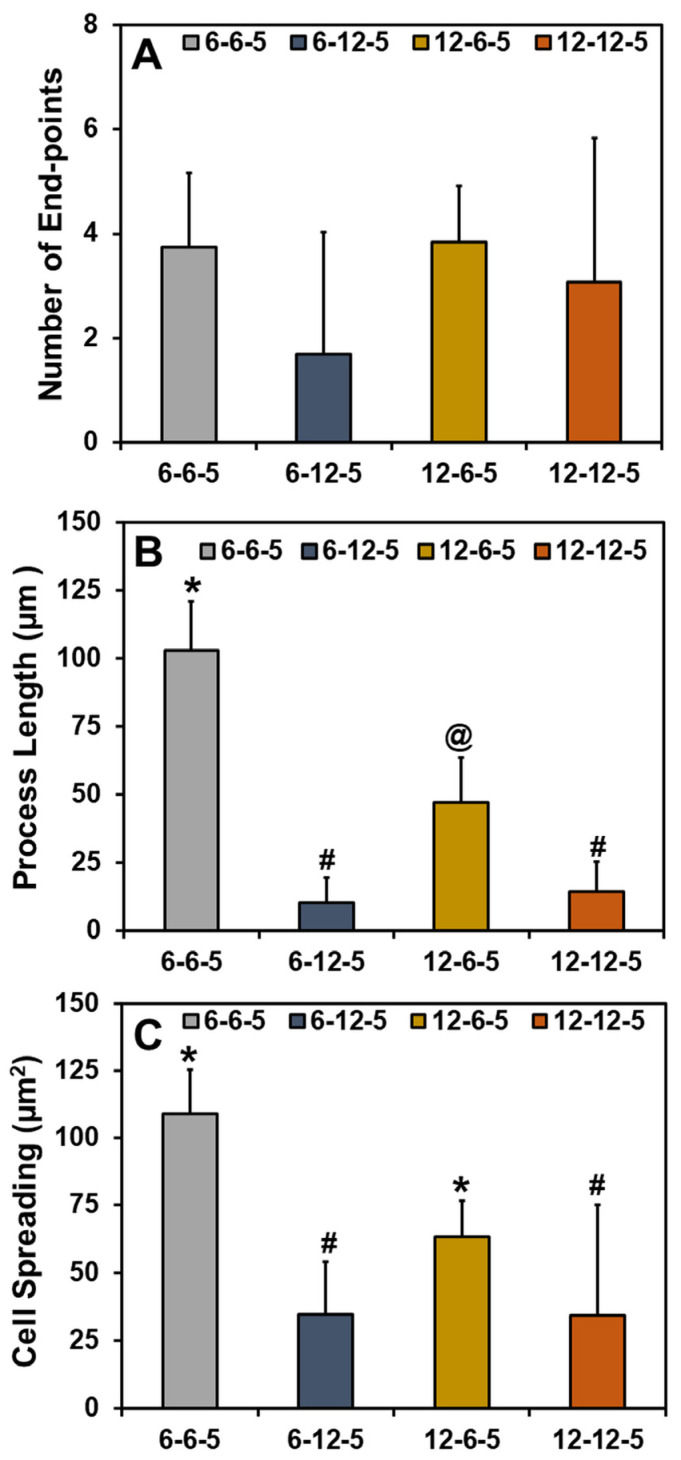
Quantitative analyses of phalloidin-stained cell cytoskeleton images for (**A**) number of end-points, (**B**) process length, and (**C**) cell spreading of NHA in ECM hydrogels. (Bars labeled with different symbols are statistically significant (*p* < 0.05)).

Quantification of the cell cytoskeleton-stained images revealed that the number of end points was comparable irrespective of the composition and stiffness of the hydrogel (Figure 6A). Results for the measurement of process lengths showed that incorporation of a higher HA amount significantly decreased (*p* < 0.05) the length of the process extension (Figure 6B). In addition, astrocytes cultured in ECM hydrogels with lower collagen content (6-6-5) exhibited significantly longer process lengths compared to 12-6-5 hydrogels, indicating that the cell process extensions are enhanced in softer hydrogels. Cell spreading quantification yielded results that agree with the visual observations seen in Figure 5 and revealed that the addition of higher HA amounts significantly decreased (*p* < 0.05) cell spreading (Figure 6C). In addition, significantly greater cell spreading (*p* < 0.05) was observed in softer hydrogels (6-6-5) compared to stiffer hydrogels (12-12-5) despite the use of the same composition of collagen and HA. Lastly, although 12-6-5 hydrogels were composed of more collagen, cell spreading was greater in 6-6-5 hydrogels compared to 12-6-5 hydrogels, indicating that astrocytes spread more in softer hydrogels. Together, these results indicate that astrocytes exhibit higher process lengths and greater spreading in softer ECM hydrogels with lower HA composition.

### 3.6. Cell Phenotype Analyses Using Immunofluorescence

**Figure 7:**
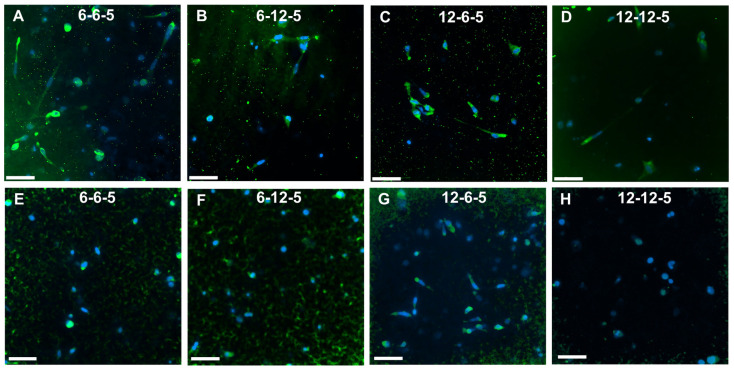
Immunofluorescence staining for assessment of cell phenotype in ECM hydrogels. (**A**–**D**) GFAP expression for reactive phenotype, and (**E**–**H**) ALDH1L1 for quiescent phenotype. Scale bar: 50 µm.

Immunofluorescence staining followed by imaging using confocal microscopy was performed to assess the effects of ECM composition and stiffness on astrocyte cell phenotype. ALDH1L1 and GFAP were used as quiescent and reactive cell phenotype markers, respectively. Cells cultured in 6-6-5 and 12-6-5 hydrogels showed prominent expression of GFAP indicating that astrocytes express a reactive phenotype (Figure 7A,C). Increasing the amount of HA (6-12-5 and 12-12-5) resulted in lower GFAP expression (Figure 7B,D). When comparing 6-6-5 and 12-12-5 hydrogels that are compositionally similar, visibly lower GFAP expression was observed in the 12-12-5 hydrogels (Figure 7A,D). Expression of ALDH1L1 appeared greater in 12-6-5 hydrogels compared to 6-6-5 hydrogels (Figure 7E,G). Changes in HA amount had no impact on ALDH1L1 expression in hydrogels with lower collagen composition (6-6-5 and 6-12-5), but ALDH1L1 expression was observed to be higher in 12-6-5 hydrogels compared to 12-12-5 hydrogels (Figure 7E–H). Together, these results suggest that both the ECM composition and stiffness can impact astrocyte cell phenotype.

## 4. Discussion

While age-related changes in brain ECM properties have been implicated in the onset and progression of neurodegenerative diseases such as AD, little is known about the role of specific ECM properties (composition, stiffness, and their interplay) on cellular level changes that may trigger the disease [11]. Although prior studies have reported on the impact of modulating the composition of specific ECM components such as HA and Matrigel on astrocytes [14,15], there is a need for a systematic investigation of the combined effects of ECM composition and stiffness on the astrocyte response to better understand the role of the ECM in brain function. Herein, xeno-free biomimetic hydrogel-based ECM models comprising collagen and HA (the backbone of the brain ECM) were employed and the impact of changes in the ECM composition and ECM stiffness on astrocyte cell morphology, proliferation, and phenotype was investigated.

Hydrogel-based ECM models employed in this study were composed of collagen and HA, two biopolymers largely found in the tissue ECM. Collagen molecules self-assemble and polymerize via thermal crosslinking to form fibers upon exposure to physiological conditions (i.e., 37 °C, pH 7.4). Polymerization of HA is mediated via PEGDA crosslinking of the thiolated functional groups, resulting in a multicomponent mIPN. Results from the current study showed that by modulating the compositions of collagen and HA it is feasible to control the stiffness of the ECM hydrogels to match the stiffness of the native brain ECM (i.e., 0.5–1 kPa [17]; Figure 1). The addition of higher amounts of PEGDA (i.e., 20 mg/mL) can help further improve the compressive modulus of the ECM hydrogels up to 2 kPa; however, PEGDA at higher concentrations was not cytocompatible. Swelling results demonstrate that collagen-rich hydrogels show significantly higher (*p* < 0.05) water absorption capability while a change in HA content had no impact on swelling ratio (Figure 2). While HA is a highly hydrophilic polysaccharide, thiol modification and crosslinking with diacrylate can reduce the number of available polar groups, increase hydrophobicity, and thereby decrease the water uptake capacity of HA-rich hydrogels.

To maintain the structural integrity of the hydrogels, a freeze-drying process was employed for sample preparation prior to SEM imaging [14]. SEM micrographs revealed that compositional changes resulted in substantial differences in the surface microstructure of the hydrogels (Figure 3). A fibrous topography typical of collagen hydrogels was only evident in softer hydrogels with the 6-6-5 composition that showed a meshed network of thick fibers (Figure 3A). Unlike prior results reported by Placone et al. that showed collagen–HA hydrogels exhibiting thick fibers and some degree of porosity [14], the results from the current study revealed that HA-rich hydrogels (6-12-5) lacked fibrous topography and were devoid of any pores (Figure 3B). This difference in outcome can be attributed to the higher concentration of HA (i.e., 12 mg/mL) incorporated within the ECM hydrogels in the current study. The sheet-like surface morphology observed in hydrogels with a higher HA composition (6-12-5, 12-12-5) has been reported in prior studies to be the characteristic appearance of HA hydrogels (Figure 3B,D) [14,18]. Hydrogels composed of higher amounts of collagen (12-6-5) showed a highly porous microstructure with median pore size ranging from 50–150 µm (Figure 3C), an expected outcome of the freeze-drying process resulting from the sublimation of ice crystals to generate a macroporous architecture [19,20].

Alamar blue assay revealed that hydrogels with higher amounts of HA (6-12-5, 12-12-5) did not support astrocyte cell growth (Figure 4). This outcome was also confirmed from the results of cell cytoskeleton staining that showed that astrocytes retain a round morphology with little to no spreading in HA-rich hydrogels (Figure 5). Previous work has reported that culturing astrocytes in pure-HA hydrogels results in rounded non-proliferative cells [14]. While pure-HA hydrogels were not employed in this work, poor cell spreading can be attributed to the lack of cell adhesion peptide sequences in ECM hydrogels with a higher HA content (6-12-5, 12-12-5) [21]. On the other hand, ECM hydrogels with a low HA content (6-6-5, 12-6-5) showed significant increases in cell metabolic activity with time (*p* < 0.05; Figure 4) and supported extensive cell spreading, which is indicative of good cell viability (Figure 5). These results agree with prior studies that employed comparable compositions of collagen and HA to form mIPN hydrogels, and suggests that collagen provides the essential cell binding sites to promote cell adhesion, spreading, and proliferation [14,15]. Apart from the ECM composition, substrate stiffness is also known to have a profound effect on cell morphology, growth, and phenotype via the modulation of the mechanosensing pathways [22]. When comparing the ECM hydrogels with lower HA content, greater cell spreading with significantly higher process lengths (*p* < 0.05) was observed in softer hydrogels (6-6-5) compared to stiffer ones (12-6-5), suggesting that changes in matrix stiffness altered cell morphology which may also be indicative of phenotypic changes in astrocytes (Figure 6).

Reactive astrogliosis is a process that leads to the activation of astrocytes in response to trauma, infection, or the onset of neurodegenerative diseases such as AD [23]. Astrocyte activation is marked by a change in cell differentiation state from quiescent to reactive phenotype and is characterized by higher GFAP expression [24]. During AD, the change in phenotype (i.e., quiescent to reactive) is known to trigger significant functional changes in astrocytes resulting in disruption in brain homeostasis, loss of synaptic function, and neuronal cell death [23,25]. Age-related changes in the stiffness of the brain ECM have been linked to cognitive decline in AD patients [10,26]. Previous work by Hu et al. using a combination of collagen and alginate hydrogels has shown that changes in matrix stiffness significantly alter astrocyte phenotype, whereby a softer matrix initiated astrogliosis, promoted greater cell spreading, and triggered the reactive phenotype in astrocytes [16]. Results from the current study agree with these findings as indicated by greater cell spreading (Figure 6), together with high GFAP expression and low ALDH1L1 expression in 6-6-5 hydrogels (Figure 7). In addition, when comparing compositionally similar ECM hydrogels (6-6-5 vs. 12-12-5), higher GFAP expression in the 6-6-5 hydrogels may be attributed to the combined effects of a low HA content and the softness of the hydrogels.

In conclusion, results from this work show that changes in ECM composition and stiffness can significantly impact astrocyte cell morphology, viability, proliferation, and phenotype. These biomimetic hydrogel-based ECM models can be further developed using blends of additional ECM components (e.g., collagen IV, laminin, fibronectin) to gain a more comprehensive understanding of the impact of the ECM on brain cell function. In addition, these models can also serve as useful tools for the identification of key ECM biomarkers and the development of novel therapeutic strategies that can attenuate the influence of ECM changes on neurodegenerative diseases such as AD.

## Figures and Tables

**Table 1:** Summary of different compositions of ECM hydrogels. Concentrations of individual components are in mg/mL.

Composition	Collagen	HA	PEGDA
Collagen = HA	6	6	5
Collagen < HA	6	12	5
Collagen > HA	12	6	5
Collagen = HA	12	12	5

## Data Availability

The data that support the findings of this study are available from the corresponding author upon reasonable request.
